# Directing Effects of Silyl and Germyl Groups in Transition‐Metal‐Catalyzed Allylic Substitution

**DOI:** 10.1002/chem.202502196

**Published:** 2025-09-13

**Authors:** Daniel Brösamlen, Martin Oestreich

**Affiliations:** ^1^ Institut für Chemie Technische Universität Berlin Straße des 17. Juni 115 10623 Berlin Germany

**Keywords:** allylic substitution, germanium, regioselectivity, silicon, transition metals

## Abstract

Transition‐metal‐catalyzed allylic substitution is a well‐established strategy for the formation of carbon–carbon and carbon–heteroatom bonds. However, most methods remain incompatible with unsymmetrical 1,3‐disubstituted allylic electrophiles as regioselectivity becomes difficult to control when the substituents are electronically and sterically similar. Achieving simultaneous control over the enantio‐ and regioselectivity as well as the alkene geometry poses an even greater challenge. To address this, sterically and electronically influential main‐group elements such as carbon's heavier homologues silicon and germanium have been strategically incorporated. These metalloids can steer the bond formation away from their proximity enabling the formation of a single regioisomer while also serving as versatile linchpins for further functionalization. This Concept summarizes the key advancements made in the field since the discovery of the allylic substitution with a particular focus on silyl‐ and germyl‐directed strategies that have expanded the synthetic utility of this highly valuable transformation.

## Graphical abstract

Despite the maturity of transition‐metal‐catalyzed allylic substitution, unsymmetrical 1,3‐disubstituted electrophiles remain underexplored due to inherent regioselectivity challenges. This Concept highlights the strategic use of silyl and germyl groups to overcome this limitation, steering bond formation with high regiocontrol and serving as linchpins for further molecular diversification.

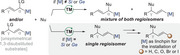

## 1 Introduction

**Scheme 1:**
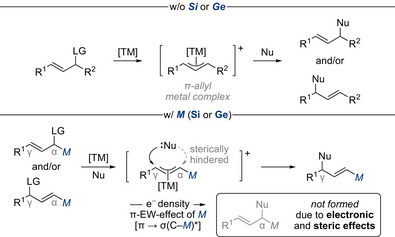
Challenges in achieving regioselectivity in transition‐metal‐catalyzed allylic substitution of unsymmetrical 1,3‐disubstituted allylic electrophiles (top). Concept of silyl and germyl groups as steering units in allylic substitution (bottom). Oxidation state of the metal center, as well as hard nucleophiles are omitted for overall clarity. EW = electron‐withdrawing. LG = leaving group. Nu = nucleophile. TM = transition metal. w/ = with. w/o = without.

Since its discovery by Tsuji in 1965^[^ ^1^ ^]^ and its subsequent development by Trost,^[^ ^2^ ^]^ transition‐metal‐catalyzed allylic substitution has emerged as a powerful and efficient strategy for the construction of C–C and C–X (X = heteroatom) bonds. These advancements have also played a central role in the development of asymmetric catalysis, enabling the efficient synthesis of enantioenriched compounds and contributing to one of the most versatile strategies for chiral transformations available to date.^[^ ^3^, ^4^ ^]^ Despite its broad utility, the use of unsymmetrical 1,3‐disubstituted allylic electrophiles remains significantly underexplored as the major challenge lies in achieving precise control over the regioselectivity as well as both the enantio‐ and diastereoselectivity. This intrinsic difficulty stems from the ambident electrophilic nature of the π‐allyl metal complex intermediate, where nucleophiles often struggle with distinguishing between two similar electrophilic termini, particularly when the substituents are electronically and sterically alike (Scheme 1, top). This results in both nucleophilic substitution (S_N_), where the nucleophile attacks at the carbon atom bearing the leaving group, and the allylic displacement (S_N_’), where the nucleophile attacks the carbon atom distal to the leaving group within the allylic system. Such competing pathways often lead to mixtures of regioisomers, complicating product isolation and downstream applications.

While directing groups covalently bound to the allylic backbone do offer a means to achieve regiocontrol, their installation and the steps required for their removal often limit their overall practicality.^[^ ^5^ ^]^ However, an effective alternative involves the use of steering groups such as carbon's higher homologues silicon and germanium which can direct the allylic substitution through a combination of electronic and steric effects (Scheme 1, bottom).^[^ ^6^ ^]^ These main‐group elements not only enhance regioselectivity but also act as versatile linchpins for further manipulations.^[^ ^7^, ^8^ ^]^

This Concept aims to illustrate the current state‐of‐the‐art in this area, highlighting the efficient directing ability of these metalloids in transition‐metal‐catalyzed allylic substitution reactions. It is organized according to the type of nucleophile involved: (1) *soft* stabilized carbon nucleophiles derived from pronucleophiles with p*K*_a_ < 25 typically resulting in overall retention of stereochemistry,^[^ ^3^, ^4^ ^]^ (2) *hard* nonstabilized carbon nucleophiles derived from pronucleophiles with p*K*_a_ > 25 generally reacting with an overall inversion of the configuration, and (3) heteroatom nucleophiles often favoring retention of stereochemistry but their reactions can also follow an inversion pathway depending on the reaction conditions and the catalyst.

## 2 Stabilized Carbon Nucleophiles

### 2.1 Utilization of Silylated Allylic Systems

**Scheme 2:**
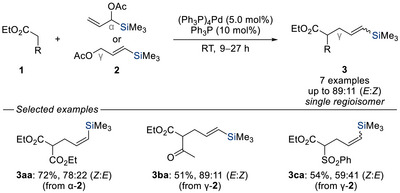
Regioselective palladium‐catalyzed allylic displacement of silylated allylic acetates with soft carbon nucleophiles (Hirao, 1981). RT = room temperature.

Since the seminal work of Tsuji and Trost on palladium‐catalyzed allylic substitution for carbon‐carbon bond formation,^[^ ^1^, ^2^ ^]^ it has evolved into a standard tool in organic synthesis. Despite its broad utility, achieving high regioselectivity had remained a longstanding challenge. A significant breakthrough came in 1981, when Hirao and co‐workers demonstrated that the regioselectivity of such transformations could be effectively influenced through the strategic incorporation of functional groups on the allylic framework (Scheme 2).^[^ ^9^ ^]^

Specifically, the use of trimethylsilyl‐substituted allylic acetates, either branched α‐**2a** or linear γ‐**2a** where α and γ refer to the position of the leaving group relative to the silyl group, in combination with soft nucleophiles under palladium catalysis led to the exclusive formation of γ‐substituted products **3aa**–**ca**. This transformation not only furnished vinylsilanes, valuable and widely employed intermediates in synthesis,^[^ ^7^, ^10^ ^]^ but also marked the first demonstration of silyl groups acting as steering units in allylic substitution. While stereochemical control over the alkene geometry was not achieved, this study laid the foundation for the use of silicon‐based groups as regioselectivity‐directing elements.

**Scheme 3:**
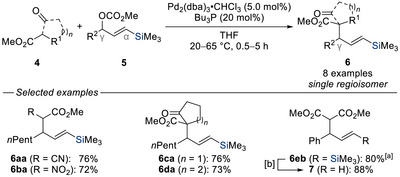
Regioselective palladium‐catalyzed substitution of silylated allylic carbonates with soft carbon nucleophiles (Tsuji, 1988 and 1989). [a] Preformed sodium dimethyl malonate was used. [b] *p*TsOH, MeCN, Δ. dba = dibenzylideneacetone. THF = tetrahydrofuran. Ts = toluenesulfonyl.

Building on these seminal insight, Tsuji and co‐workers further advanced the palladium‐catalyzed regioselective allylic substitution in a series of studies published in 1988 and 1989 (Scheme 3).^[^ ^11^ ^]^ In these efforts, a variety of acyclic and cyclic soft carbon nucleophiles **4** were employed with unsymmetrical 1,3‐disubstituted allylic carbonates **5** bearing a trimethylsilyl substituent as a steering unit. These reactions consistently furnished the vinylsilanes **6** as a single regioisomer. Notably, the observed selectivity could not be solely attributed to the steric bulk of the silyl group as substrate **5b** bearing a comparably bulky phenyl substituent still yielded the vinylsilane product **6eb**, underscoring the unique electronic and conformational influence of the silyl group. Furthermore, facile protodesilylation of vinylsilane **6eb** under acid‐catalyzed conditions furnished the corresponding terminal alkene **7** in 88% yield.

**Scheme 4:**
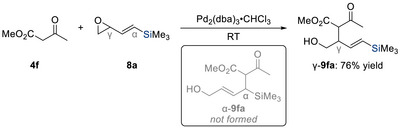
Regioselective palladium‐catalyzed substitution of silylated allylic epoxide **8a** with methyl acetoacetate **4f** (Tsuji, 1988 and 1989).

Additionally, in the context of vinyl epoxides, palladium‐catalyzed allylic substitution with carbon nucleophiles typically favors S_N_’ products, corresponding to the conventional regioselectivity.^[^ ^12^ ^]^ However, when a trimethylsilyl group was attached to the alkene terminus as in **8a**, a pronounced steering effect reversed this trend, instead leading to the formation of the uncommon γ‐**9fa** (Scheme 4). A similar phenomenon was later observed in 1999 by Salaün and co‐workers with cyclopropyl‐substituted systems where the presence of a silyl group was able to override the inherent ring strain of the three‐membered ring, promoting direct substitution at the cyclopropyl moiety (not shown).^[^ ^13^ ^]^

**Scheme 5:**
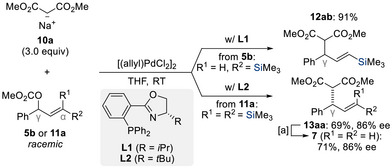
Enantio‐ and regioselective palladium‐catalyzed substitution of bis(silylated) allylic carbonate **11a** with sodium dimethylmalonate **10a** (Romero, 1997). [a] *p*TsOH, MeCN, Δ. ee = enantiomeric excess.

**Figure 1:**
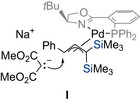
Proposed steric model rationalizing the asymmetric induction of the product formation of **13aa** (Romero, 1997).

Allylic substitution has profoundly influenced the development of asymmetric synthesis, enabling access to architecturally complex chiral molecules.^[^ ^3^, ^4^ ^]^ In 1997, Romero investigated the first enantioselective transformation of silylated allylic electrophiles with soft carbon nucleophiles, aiming to preserve the regiocontrol imparted by the silyl group (Scheme 5).^[^ ^14^ ^]^ Under palladium catalysis employing the Phox ligand **L1**, the reaction of monosilylated allylic carbonate **5b** with dimethyl sodium malonate **10a** delivered the γ‐substituted product **12ab** in 91% yield, yet without enantioinduction. Conversely, when the team employed bis(silylated) allylic carbonate **11a** combined with **L2**, the reaction afforded the vinylsilane **13aa** in 69% yield with an enantiomeric excess of 86% as a single regioisomer (for a steric model see Figure 1). Subsequent treatment of **13aa** with *p*‐toluenesulfonic acid cleanly yielded the protodesilylated terminal alkene **7** without racemization. This strategy was further applied in the structure‐based design of HIV protease inhibitors, highlighting the broader relevance of silyl‐directed, enantioselective allylic substitution.^[^ ^15^ ^]^

**Scheme 6:**
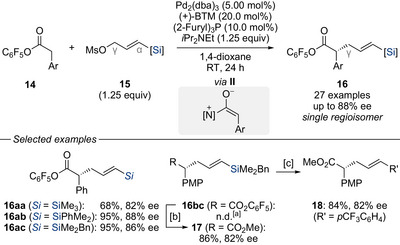
Enantio‐ and regioselective palladium‐catalyzed substitution of silylated allylic mesylates with prochiral phenylacetic acid esters (Snaddon, 2018). [a] **16bc** was formed in situ and processed without any further purification. [b] DMAP (20 mol%), Et_3_N (5.0 equiv), MeOH, 65 °C for 18 hours. [c] Pd_2_(dba)_3_ (2.5 mol%), TBAF (2.2 equiv), H_2_O (3.0 equiv), *p*CF_3_C_6_H_4_–I (1.5 equiv), THF, 0 °C to RT for 24 hours. Ar = aryl. BTM = benzotetramisole. DMAP = 4‐dimethylaminopyridine. n.d. = not determined. PMP = *p*‐methoxyphenyl. TBAF = tetrabutylammonium fluoride.

Almost two decades later, Snaddon and co‐workers introduced a strategically distinct approach to achieve enantio‐ and regioselective palladium‐catalyzed allylation of linear silylated allylic mesylates **15** with prochiral phenylacetic acid esters **14** (Scheme 6).^[^ ^16^ ^]^

In contrast to Romero's method employing geminal bis(silylated) allylic carbonate,^[^ ^14^ ^]^ this strategy utilizes C1‐ammonium enolates **II**, stereodefined ester enolate equivalents, to facilitate the formation of the stereogenic centre. The corresponding vinylsilanes **16** were obtained as a single regioisomer with good enantioselectivity across a range of substrates. Its synthetic versatility was further demonstrated through a Hiyama cross‐coupling which delivered the functionalized product **18** in high yield without erosion of the stereocenter.

**Scheme 7:**
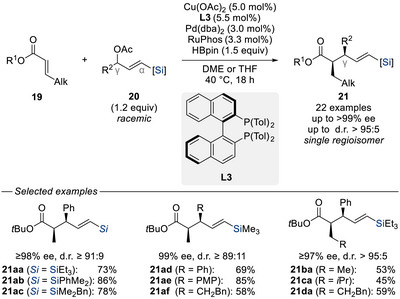
Enantio‐ and regioconvergent hydroallylation of acrylates with silylated allylic acetates under dual copper/palladium catalysis (Suzuki and Matsuda, 2025). Alk = alkyl. DME = 1,2‐dimethoxyethane. d.r. = diastereomeric ratio. RuPhos = 2‐dicyclohexylphosphino‐2′,6′‐diisopropoxybiphenyl. Tol = tolyl.

**Scheme 8:**
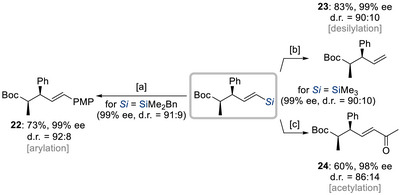
Silicon as a linchpin for further derivatizations (Suzuki and Matsuda, 2025). [a] Pd_2_(dba)_3_ (2.5 mol%), TBAF (2.0 equiv), PMP–I (1.5 equiv), THF, RT for 1 hour. [b] TsOH•H_2_O (1.0 equiv), MeCN, 90 °C for 3 hours, then Me–I (3.0 equiv), K_2_CO_3_ (3.0 equiv), DMF, RT for 2 hours. [c] [RhCl(CO)_2_]_2_ (5.0 mol%), Ac_2_O (3.0 equiv), 1,4‐dioxane, 90 °C for 48 hours. Boc = *tert*‐butyloxycarbonyl.

In a recent advancement, Suzuki and Matsuda reported a stereodivergent strategy for the simultaneous construction of two adjacent stereocenters by leveraging the silyl group as a regioselectivity‐directing unit (Scheme 7).^[^ ^17^ ^]^ The key to the success of this enantio‐ and regioconvergent formal hydroallylation of acrylates **19** with racemic silylated allylic acetates **20** was the use of a dual copper/palladium catalytic system, enabling the formation of vicinal stereocenters in the resulting vinylsilanes **21**. The reaction exhibited excellent levels of enantio‐ and diastereoselectivities for various substrates, highlighting the power of silicon as a steering group in overcoming regioselectivity challenges typically associated with allylic substitution. Moreover, the synthetic utility of the vinylsilane products was demonstrated, including the arylation (**21ac** → **22**), protodesilylation (**21ad** → **23**), and acetylation (**21ad** → **24**) all of which proceed without racemization (Scheme 8).

### 2.2 Mechanistic Insights into the Silicon's Steering Effect

To gain deeper insight into the fundamental role of silicon's steering effect in allylic substitution, Pleixats and co‐workers conducted a theoretical investigation using DFT methods in 2002.^[^ ^6^ ^]^ Optimized geometries of silylated allylpalladium cations **III**–**V** revealed two principal electronic factors explaining the steering effect of the silicon unit (Table 1). First, the Pd–C3 bond is shortened due to the electron‐withdrawing effect of the silicon atom via π–σ*(C–Si) orbital interaction. Second, silicon's lower electronegativity relative to carbon induces a substantial negative charge on the allyl carbon atom directly bound to the silicon atom, creating a pronounced charge asymmetry between the termini C1 and C3 of the allyl group. These factors together shorten the Pd–C3 bond, which ultimately determines the observed regioselectivity. Furthermore, the energy barriers for the nucleophilic attack of ammonia at either of the terminal allyl carbon atoms in silyl‐substituted bis(phosphino)(η^3^‐allyl)palladium complexes were calculated, revealing that bulky silyl groups direct nucleophiles to the carbon distal to the silicon atom through combined steric and electronic effects. In turn, less sterically demanding silyl groups render the α‐position more accessible (C3) while electronic factors still favor the attack at the distal position (C1). This observation is in agreement with experimental findings by Trost, who emphasized that electronic effects, particularly charge distribution within the π‐allyl complex, play a more dominant role than sterics in dictating the regioselectivity.^[^ ^18^ ^]^ Interestingly, earlier experimental work by Murahashi in 1985 had already shown that alkyl substituents are more sterically demanding than silicon analogues.^[^ ^19^ ^]^ This supports the view that silicon is less electron‐donating relative to alkyl or phenyl substituents located on the opposite end of the allyl moiety.^[^ ^6^ ^]^ In turn, in cases involving more strongly electron‐withdrawing groups (EWGs) such as esters, nucleophilic substitution preferentially occurs at the carbon bonded to the silicon atom, effectively overriding the silyl‐induced regioselectivity (not shown).^[^ ^20^ ^]^ It is important to note that this characteristic becomes pronounced when an allylic silicon group is present, as silicon in this position functions as an electron‐donating group due to orbital interaction between the σ(C–Si) bond and the LUMO (d_π_*) of the allyl–palladium fragment.^[^ ^21^ ^]^

**Table 1:** Theoretical studies on the role of silicon in palladium‐catalyzed allylic substitution (Pleixats, 2002). Selected geometry parameters and charge distribution for silylated allylpalladium complexes (**III**–**V**).

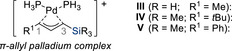							
	Bond lengths [Å]		Charge densities [au]				
Complex	Pd–C1	Pd–C3	C1	C2	C3	Pd	(H_3_P)_2_Pd
**III**	2.29	2.25	−0.21	−0.20	−0.71	0.29	0.86
**IV**	2.34	2.25	−0.20	−0.22	−0.72	0.29	0.81
**V**	2.32	2.27	−0.21	−0.21	−0.73	0.28	0.80

### 2.3 Utilization of Silylated Bis(allylic) Systems

**Scheme 9:**
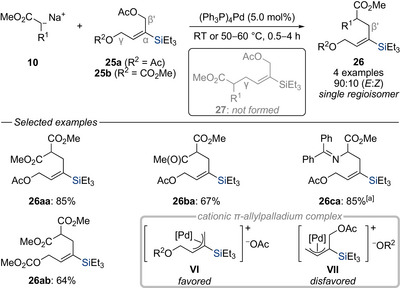
Chemo‐ and regioselective palladium‐catalyzed substitution of silylated allylic bis(electrophiles) with soft carbon nucleophiles (Malacria, 1996). [a] Isolated with *E*:*Z* = 70:30.

**Figure 2:**
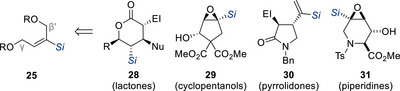
Silylated bis(allylic) derivatives as synthons for the stereoselective synthesis of highly substituted scaffolds. El = electrophile.

Malacria and co‐workers initiated studies on the influence of silicon when attached to the central position of cationic π‐allylpalladium complexes (Scheme 9).^[^ ^22^ ^]^ The precursors **25** feature two potential leaving groups that differ only in their position relative to the silicon atom. When silylated allylic bis(acetate) **25a** was employed with sodium dimethylmalonate **10a** under palladium catalysis, the resulting allylated products **26aa**–**ca** were obtained with excellent chemo‐ and regioselectivities. The high level of chemoselectivity observed experimentally was further corroborated by theoretical calculations,^[^ ^23^ ^]^ demonstrating that the π‐allylpalladium cation complex **VI** is more stabilized than isomeric **VII**. The primary reason for the observed effect is attributed to the stronger palladium–allyl interaction when the silicon atom occupies the central carbon of the π‐allylpalladium cation complex (**VI**). The authors also point out a possible analogy, though not necessarily a mechanistic correlation, to the known stabilization of carbocations by silicon located in the β‐position.^[^ ^24^ ^]^ This explains why the acetate group γ to the silyl substituent remains intact (**26aa**–**ca**) and why the acetate is preferentially displaced over the carbonate despite the latter typically being a better leaving group (**25b** → **26ab**). Additional studies replacing the silyl by a *tert*‐butyl group revealed that the reaction still followed the same chemoselective pathway, although with significantly reduced stereoselectivity for the alkene geometry (not shown).^[^ ^25^ ^]^ These findings further highlight silicon's dual influence of both steric and electronic effects in governing the chemo‐ and stereoselectivity of this transformation. Malacria and co‐workers subsequently extended the use of these silylated allylic bis(acetates) further and applied these synthons to the stereoselective synthesis of highly substituted lactones,^[^ ^26^ ^]^ cyclopentanols,^[^ ^27^ ^]^ pyrrolidones,^[^ ^28^ ^]^ and piperidines^[^ ^29^ ^]^ (Figure 2).

## 3 Nonstabilized Carbon Nucleophiles

**Scheme 10:**
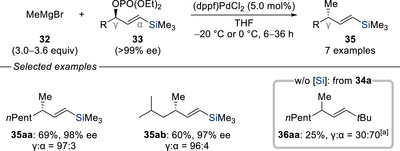
Regioselective and enantiospecific palladium‐catalyzed allylic substitution of silylated allylic phosphates with a Grignard reagent (Sato, 1993). [a] Racemic form of **34a** was used. dppf = 1,1′‐bis(diphenylphosphino)ferrocene.

The use of soft carbon nucleophiles in palladium‐catalyzed allylic substitution is well‐established. In contrast, the application of hard carbon nucleophiles, especially within a palladium catalytic system, has been comparatively less explored. In 1993, Sato and co‐workers reported the first palladium‐catalyzed allylic displacement of enantioenriched silylated allyl phosphates **33** with Grignard reagent **32**, achieving complete regiocontrol in a stereospecific manner to access stereogenic γ‐chiral vinylsilanes (Scheme 10).^[^ ^30^ ^]^ The observed inversion of configuration in the products aligns with the expected stereochemical course of allylic substitutions involving hard nucleophiles.^[^ ^3^, ^4^ ^]^ To probe the influence of silicon's steering effect, the silyl group was replaced with a *tert*‐butyl group **34a**, a sterically demanding analogue expected to determine the regioselectivity solely based on bulk. However, this modification resulted in a significant drop in regioselectivity (γ:α = 30:70), underscoring that electronic factors rather than steric hindrance alone play a pivotal role. This finding lends further support of the experimental and computational evidence previously discussed.^[^ ^6^, ^18^ ^]^

**Scheme 11:**
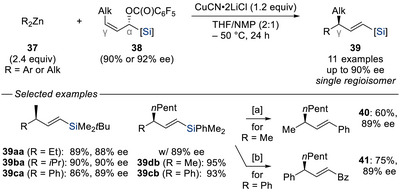
Diastereo‐ and regioselective copper‐catalyzed *anti*‐S_N_2’ allylic displacement of enantioenriched α‐pentafluorobenzoyl allylic silanes with dialkyl‐ and diarylzinc reagents (Knochel, 2007). [a] BCl_3_ (4.0 equiv), CH_2_Cl_2_, −30 °C for 5 hours, then (Ph_3_P)_4_Pd (5.0 mol%), Na_2_CO_3_ (2.0 equiv), Ph–I (1.0 equiv), toluene, 100 °C for 3 hours. [b] AlCl_3_ (1.2 equiv), BzCl (1.2 equiv), CH_2_Cl_2_, −78 °C to RT for 3 hours. NMP = *N*‐methyl‐2‐pyrrolidone.

Beyond palladium catalysis, first‐row transition metals^[^ ^31^ ^]^ such as copper^[^ ^32^, ^33^ ^]^ and nickel^[^ ^34^ ^]^ have emerged as attractive alternatives in allylic substitution reactions, not only due to their abundance and cost but also because of their compatibility with hard nucleophiles. In 2007, Knochel and co‐workers leveraged the silicon's steering effect in a copper‐catalyzed, diastereo‐ and regioselective *anti*‐S_N_2’ allylic displacement of enantioenriched α‐pentafluorobenzoyl‐substituted allylic silanes **38** with dialkyl‐ and diarylzinc reagents **37** (Scheme 11).^[^ ^35^ ^]^ This transformation delivered the desired vinylsilanes **39** in good yields and with high levels of enantioselectivities. Importantly, the products also served as versatile building blocks as demonstrated by their borodesilylation (= Si–B exchange) followed by a Suzuki–Miyaura cross‐coupling (**39db** → **40**) as well as their Friedel–Crafts acylation (**39cb** → **41**) with preservation of the stereochemical information in both cases.

**Scheme 12:**
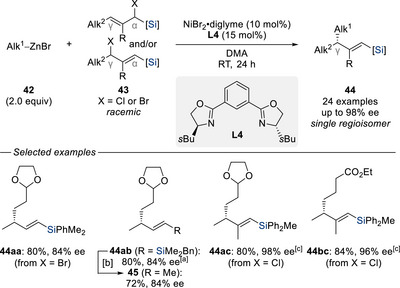
Enantio‐ and regioconvergent nickel‐catalyzed substitution of silylated allylic halides with alkylzinc nucleophiles (Oestreich, 2021). [a] Silylated allylic bromide **43b** was used. [b] CuI (1.5 equiv), (EtO)_3_P (1.5 equiv), Bu_4_NF•(*t*BuOH)_4_ (2.4 equiv), MeI (3.0 equiv), DMF, RT for 24 hours. [c] NiI_2_ was used instead of NiBr_2_•diglyme. diglyme = bis(2‐methoxyethyl) ether. DMA = dimethylacetamide.

Nickel catalysis has garnered increasing attention in recent years due to its unique reactivity profile with several advantages over other transition metals.^[^ ^36^ ^]^ Among the transformations explored, allylic substitution has emerged as a promising domain for nickel catalysis.^[^ ^34^ ^]^ In line with our ongoing interest in silicon‐based methodology development utilizing nickel catalysts,^[^ ^37^ ^]^ we investigated in 2021 the directing ability of silyl groups in nickel‐catalyzed allylic substitution, culminating in the development of an enantio‐ and regioconvergent method for the allylation of primary alkylzinc reagents **42** with regioisomeric mixtures of racemic α/γ‐silylated^[^ ^38^ ^]^ allylic halides **43** (Scheme 12).^[^ ^39^ ^]^ This approach afforded the enantioenriched γ‐chiral vinylsilanes **44aa**–**ac** and **44bc** in good yields and with high enantioselectivities. Notably, the silyl group played a crucial role in overriding the inherent lack of selectivity which had previously been observed when a simple primary alkyl‐substituted allylic electrophile was used under similar conditions.^[^ ^40^ ^]^ Again, the silyl group offered an opportunity to further derivatize, enabling further carbon–carbon bond formation without compromising the stereochemical integrity (e.g., **44ab** → **45**).

**Scheme 13:**
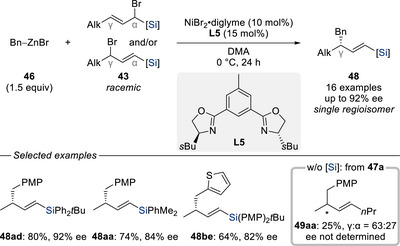
Enantio‐ and regioconvergent nickel‐catalyzed substitution of silylated allylic bromides with benzylzinc nucleophiles (Oestreich, 2022).

Building on our earlier work, we subsequently expanded this methodology to benzylzinc nucleophiles (Scheme 13),^[^ ^41^ ^]^ which are attractive due to the synthetic relevance of benzyl motifs and the well‐documented challenges associated with their use as nucleophiles in cross‐coupling transformations, most notably, their tendency to undergo Wurtz‐type homocoupling. A variety of benzylzinc reagents **46** were subjected to the enantio‐ and regioconvergent allylic substitution with regioisomeric mixtures of racemic α/γ‐silylated^[^ ^38^ ^]^ allylic bromides **43**, delivering the corresponding products **48** in good yields along with high enantioselectivities. As anticipated, replacing the silyl directing group with a simple primary alkyl substituent resulted in a complete loss of the regioselectivity (**47a** → **49aa**).

**Scheme 14:**
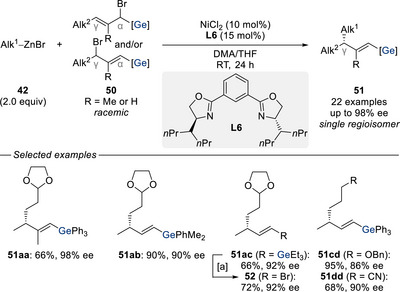
Enantio‐ and regioconvergent nickel‐catalyzed substitution of germylated allylic bromides with alkylzinc nucleophiles (Oestreich, 2023). [a] NBS (2.0 equiv), DMF, RT for 2 h. NBS = *N*‐bromosuccinimide. DMF = dimethylformamide.

Unlike carbon and silicon, the chemistry of organogermanes remains largely underexplored, despite their several advantages such as chemical robustness, low toxicity, and versatile reactivity.^[^ ^8^ ^]^ Motivated by this, we had decided to extend our previous work^[^ ^39^, ^41^ ^]^ by investigating the related steering effect by germyl groups in nickel‐catalyzed allylic substitution reactions (Scheme 14).^[^ ^42^ ^]^ When racemic α/γ‐germylated allylic bromides **50** were subjected to nickel catalysis with alkylzinc bromides **42**, the desired γ‐chiral vinylgermanes **51aa**–**ac** and **51cd**,**dd** were obtained in an enantio‐ and regioconvergent fashion. While the high enantioselectivities were induced by the newly designed Pybox ligand **L6**, the regioselectivity was governed by the germyl group. Moreover, the resulting vinylgermanes proved synthetically highly versatile as demonstrated by successful bromodegermylation (**51ac** → **52**) and subsequent cross‐coupling transformations, all without erosion of stereochemical integrity (not shown).

## 4 Heteroatom Nucleophiles

### 4.1 Nitrogen Nucleophiles

**Scheme 15:**
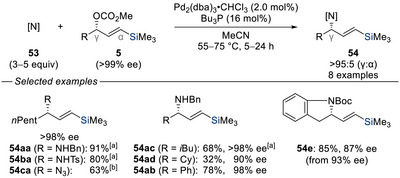
Enantiospecific and regioselective amination of silylated allylic carbonates with N‐nucleophiles for the preparation of enantioenriched y‐silylallylamines (Sato, 1993). [a] Average yield shown. [b] Reaction performed with the palladium catalyst (2.0 mol%) and Bu_3_P (8.0 mol%) in THF/H_2_O (2:1).

Aside from carbon nucleophiles, the use of heteroatom nucleophiles in allylic substitution reactions has enabled access to a broad range of valuable building blocks. In particular, the incorporation of nitrogen nucleophiles by allylic substitution reactions to access chiral amines was central to the total synthesis of several natural products.^[^ ^43^ ^]^ However, the use of unsymmetrical 1,3‐disubstituted allylic electrophiles in such transformations has been far less explored, largely due to the regioselectivity challenges associated with these substrates. In 1993, Sato reported the preparation of chiral allylic amines utilizing the silyl group to govern the regioselectivity (Scheme 15).^[^ ^44^ ^]^ Enantioenriched trimethylsilyl‐substituted allylic carbonates **5** were reacted with nitrogen nucleophiles **53** under palladium catalysis to afford the corresponding γ‐silylated allylic amines **54aa**–**ca**, **54ab**–**ad**, and **54e** with retention of the enantiopurity. This suggests that nitrogen nucleophiles behave as soft nucleophiles under these conditions. Furthermore, intramolecular allylic amination was also demonstrated, as exemplified by the transformation of substrate **5e** → **54e**.

**Scheme 16:**
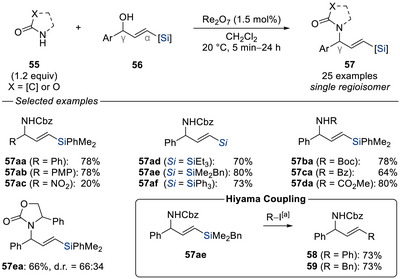
Regioselective rhenium‐catalyzed allylic amination of silylated allylic alcohols with carbamates (Cook, 2014). [a] Reaction performed with Pd_2_(dba)_3_•CHCl_3_ (5.0 mol%), TBAF (2.2 equiv), R–I (1.0 equiv), 1,4‐dioxane, 20 °C. Cbz = benzyloxycarbonyl.

In 2014, Cook and co‐workers reported a rhenium‐catalyzed allylic amination utilizing carbamates as nucleophiles, where a silyl group is crucial for achieving high levels of regioselectivity (Scheme 16).^[^ ^45^ ^]^ γ‐Silylated allylic alcohols **56** reacted with carbamates **55** under rhenium catalysis to afford the corresponding aminated products **57** in good to excellent yields. Notably, the silyl group enabled both C(sp^2^)–C(sp^2^) and C(sp^2^)–C(sp^3^) Hiyama cross‐couplings (**57ae** → **58** and **57ae** → **59**, respectively).

**Scheme 17:**
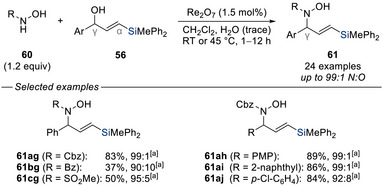
Regioselective rhenium‐catalyzed allylic amination of silylated allylic alcohols with *N*‐hydroxycarbamates and *N*‐hydroxysulfonamides as nitrogen nucleophiles (Cook, 2014). [a] *N*:*O* ratio.

At the same time, this laboratory expanded the methodology by employing a broader range of nitrogen nucleophiles, including *N*‐hydroxycarbamates and *N*‐hydroxysulfonamides (Scheme 17).^[^ ^46^ ^]^ These reactions not only demonstrated excellent *N*,*O*‐chemoselectivity but also maintained high regioselectivity in the allylic displacement itself.

### 4.2 Oxygen Nucleophiles

**Scheme 18:**
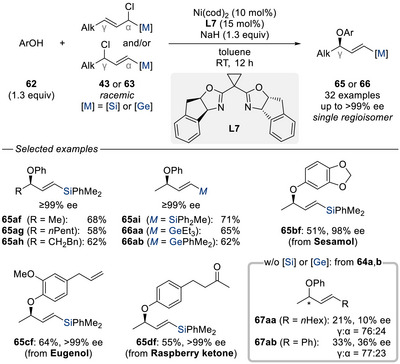
Enantio‐ and regioconvergent nickel‐catalyzed allylation of phenol derivatives with silylated‐ and germylated allylic chlorides (Oestreich, 2024). cod = cycloocta‐1,5‐diene.

**Scheme 19:**
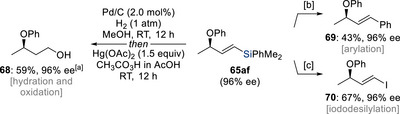
Silicon as a linchpin for further functionalization (Oestreich, 2024). [a] Overall yield from two steps. [b] (Ph_3_P)_2_PdCl_2_ (5.0 mol%), Ph_3_P (10 mol%), CuI (1.0 equiv), TBAF (3.0 equiv), Ph–I (1.2 equiv), DMF, 100 °C for 15 hours. [c] NIS (3.0 equiv), MeCN, 60 °C for 15 hours. NIS = *N*‐iodosuccinimide.

Oxygen nucleophiles have long been employed in transition‐metal‐catalyzed allylic substitution reactions, particularly in asymmetric methodologies, as the resulting allylic alcohol motif is found in countless bioactive compounds.^[^ ^3^, ^47^ ^]^ However, the vast majority of reported methods rely on branched or linear allylic electrophiles that proceed through a terminal π‐allyl intermediate^[^ ^3^, ^48^ ^]^ due to the challenges associated with precise control over both enantio‐ and regioselectivity as well as the alkene geometry in unsymmetrical 1,3‐disubstituted allylic electrophiles. Based on our previous work on nickel‐catalyzed enantio‐ and regioconvergent C(sp^3^)–C(sp^3^) cross‐coupling strategies,^[^ ^39^, ^41^, ^42^ ^]^ we developed a method for the synthesis of chiral allylic aryl ethers (Scheme 18).^[^ ^49^ ^]^ With phenol derivatives **62** and silylated or germylated allylic chlorides **43** or **63**, the reaction proceeded under nickel catalysis with excellent enantioselectivities to afford γ‐chiral vinylmetalloids **65** or **66**. To illustrate the pivotal role of the metalloid substituents, we examined two allylic chlorides, one bearing a simple alkyl chain as in **64a** and another an aryl group as in **64b** in place of the silyl or germyl moiety. Under otherwise identical conditions, both enantioselectivity and regioselectivity deteriorated significantly, and the desired allylic aryl ethers **67aa** and **67ab** were obtained in poor yields, clearly demonstrating the critical steering effect imparted by the metalloid groups. Beyond the regio‐ and stereoselective formation of the products further racemization‐free functionalizations were explored (Scheme 19). These included a 1,3‐chirality transfer via a Claisen rearrangement, deprotection to access the chiral free allylic alcohol (not shown), and leveraging the silyl group for facile oxidation (**65af** → **68**), Hiyama coupling (**65af** → **69**), and iododesilylation (**65af** → **70**).

## 5 Summary and Outlook

In this concept, we summarized the progress made since the pioneering work of Tsuji and Trost on directed allylic substitution. We focused on the strategic use of main‐group elements, specifically silicon and germanium, to control the regioselectivity in challenging allylic displacement of unsymmetrical 1,3‐disubstituted allylic electrophiles. Both theoretical calculations and experimental studies have shown that, aside from sterics, electronic effects do also play a significant role in governing the regioselectivity with a silicon atom at the allyl terminus. Comparable mechanistic studies involving germanium‐containing analogues have, to the best of our knowledge, not yet been reported. We highlighted the current scope of compatible nucleophiles, including *soft* and *hard* carbon as well as heteroatom nucleophiles, and demonstrated how these metalloids serve as versatile linchpins for further functional‐group manipulations, enabling the formation of C–H, C–C, C–O, C–Br, and C–I bonds. We believe this strategy continues to offer great potential for synthesis of densely functionalized molecular architectures where regiochemical issues need to be solved. Ongoing efforts in our laboratory are directed toward expanding this methodology to other heteroatom nucleophiles.

## Conflict of Interest

The author declares no conflicts of interst.

## Data Availability

Data sharing is not applicable to this article as no new data were created or analysed in this study.
